# TIPRL Inhibits Protein Phosphatase 4 Activity and Promotes H2AX Phosphorylation in the DNA Damage Response

**DOI:** 10.1371/journal.pone.0145938

**Published:** 2015-12-30

**Authors:** Kimberly Romero Rosales, Michael A. Reid, Ying Yang, Thai Q. Tran, Wen-I Wang, Xazmin Lowman, Min Pan, Mei Kong

**Affiliations:** Department of Cancer Biology, Beckman Research Institute of City of Hope Cancer Center, Duarte, California, United States of America; The University of Hong Kong, HONG KONG

## Abstract

Despite advances in our understanding of protein kinase regulation in the DNA damage response, the mechanism that controls protein phosphatase activity in this pathway is unclear. Unlike kinases, the activity and specificity of serine/threonine phosphatases is governed largely by their associated proteins. Here we show that Tip41-like protein (TIPRL), an evolutionarily conserved binding protein for PP2A-family phosphatases, is a negative regulator of protein phosphatase 4 (PP4). Knockdown of TIPRL resulted in increased PP4 phosphatase activity and formation of the active PP4-C/PP4R2 complex known to dephosphorylate γ-H2AX. Thus, overexpression of TIPRL promotes phosphorylation of H2AX, and increases γ-H2AX positive foci in response to DNA damage, whereas knockdown of TIPRL inhibits γ-H2AX phosphorylation. In correlation with γ-H2AX levels, we found that TIPRL overexpression promotes cell death in response to genotoxic stress, and knockdown of TIPRL protects cells from genotoxic agents. Taken together, these data demonstrate that TIPRL inhibits PP4 activity to allow for H2AX phosphorylation and the subsequent DNA damage response.

## Introduction

Phosphorylation of histone H2A variant H2AX is one of the earliest events that occur in response to DNA double stranded breaks (DSB). H2AX phosphorylation at Ser 139 is mediated by members of the PI(3)K (phosphatidyl-inositol-3-OH kinase)-like kinases including ATM (ataxia telanglectasia), ATR (ATM- and Rad3-related), and DNA-PK (DNA-dependent protein kinase) to generate γ-H2AX. Phosphorylation of H2AX results in recruitment and accumulation of DNA repair proteins needed to mediate DNA repair and checkpoint signaling [1,2].

Although, the kinases that regulate the formation of γ-H2AX are well understood, much less is known about the regulation of protein phosphatases, which mediate dephosphorylation of γ-H2AX and are required for recovery from DNA damage. Recent studies have shown that the PP2A family protein phosphatase, PP4, acts as a γ-H2AX phosphatase [3]. However, unlike kinases, protein phosphatases usually recognize their substrate specificity through assembly into distinct complexes. For example, a deletion screen in yeast revealed that a trimeric complex, the histone H2A phosphatase complex containing PPH3, PSY2, and YB1046W regulates the phosphorylation status of γ-H2AX *in vivo* [4]. PP4-C is the closest human homolog of yeast *Pph3* and studies elucidating the role of PP4 in mammalian cells found that, similar to yeast, a complex containing PP4R2 (YB1046W in yeast) and PP4R3β (PSY2 in yeast) control cellular levels of γ-H2AX [5–7]. Knockdown of PP4-C or PP4R2 resulted in hypersensitivity to DNA replication inhibitors including camptothecin (CPT) and hydroxyurea [6]. In addition, we have shown that suppression of PP2A-family phosphatases resulted in hyperphosphorylation of H2AX [8].

TIPRL is an evolutionarily conserved protein and was first identified in yeast to interact with Tap42 (α4) to regulate protein phosphatase activity [9]. In mammalian cells, TIPRL does not directly bind α4, but rather primarily interacts with PP2A, PP4 or PP6 catalytic subunits [10–13]. However, the consequence of TIPRL’s association with the C subunit in phosphatase biology and cell signaling is unclear. In this study, we found that TIPRL interacts with PP4R2 and PP4R3β, both regulatory subunits of the PP4 complex shown to de-phosphorylate γ-H2AX [6]. Similar to the yeast phenotype, TIPRL depleted cells were protected from DNA damage-induced death, while TIPRL overexpressing cells were sensitized to the DNA damaging agents CPT and doxorubicin. Taken together, these studies reveal that TIPRL plays a critical role in regulation of γ-H2AX levels by inhibiting protein phosphatase activity.

## Material and Methods

### Cell culture and cell death assay

3T3 MEFs, 293Ts and HeLa cells (ATCC) were cultured in Dulbecco’s modified Eagle’s medium (DMEM) containing 25mM Glucose and 4mM L-Glutamine supplemented with 10% (v/v) fetal bovine serum (FBS) (FBS, Gemini BioProducts), 100 units/mL of penicillin, and 100 μg/mL of streptomycin. For doxorubicin (Sigma), and CPT (Sigma) treatment, drugs were added to the medium at the indicated doses. At the desired time points, cells were collected by trypsinization and incubated with propidium iodide (PI, 1 μg/mL; Molecular Probes). Cell death was determined using flow cytometry by PI exclusion. To examine recovery following exposure to drugs, cells were treated with CPT for 1.5 h, then washed and fed with fresh medium with no drugs, and cultured for the indicated periods of time. For the colorimetric MTS-PMS assay (Promega), cells were seeded at 10,000 cells/well in a 96-well plate followed by doxorubicin treatment at the indicated concentrations for 24 hours. 20 μl of MTS/PMS solution (final concentrations 333 μg/ml MTS and 25 μM PMS) were added to 100 μl of media per well. Cells were incubated with the MTS-PMS reagent for 1hr at 37°C. Absorbance was measured at 490nm with a spectrophotometer.

### Immunoblotting and immunofluorescence

Cells were lysed in RIPA buffer (1% sodium deoxycholate (v/v), 0.1% SDS (v/v), 1% Triton X-100 (v/v), 10 mM Tris at pH 8.0, 150 mM NaCl) with protease inhibitor complex (Roche). Equal amounts of protein (10–40 μg) were loaded on precast 4%–12% Bis-Tris NuPAGE gels (Invitrogen), followed by transfer onto nitrocellulose. Immunoblotting was performed with the following antibodies: FLAG (M2), tubulin, and β-actin (Sigma); p-H2AX S139 (γ-H2AX), cleaved caspase 3, PARP (Cell Signaling); PP2A-C subunit (1D6, Upstate); PP2A-A subunit (α and β isoforms) (6G3, Cell Signaling; or sheep, Abcam); TIPRL, PP4-C, PP4R2 (Bethyl). For immunofluorescence microscopy, cells were plated on 12mm coverslips coated with poly-L-lysine D. Cells were treated with 5uM CPT for 1hr then fixed in 4% (v/v) paraformaldehyde and permeabilized for 10 min in PBS containing 0.2% (v/v) Triton X-100. Cells were washed with PBS containing 0.02% (v/v) Triton X-100 and 1.5% (v/v) FBS, followed by incubation with anti-γ-H2AX antibody (Cell Signaling) for 1 h at room temperature. Cells were incubated with Alexa Fluor (594)-conjugated secondary antibody (Molecular Probes). Nuclei were visualized by staining with 1 μg/mL DAPI. Images were captured using an Olympus BX50 upright microscope Hammamatsu Orca high speed/ high resolution digital camera at 60x objective and images were analyzed using Image-Pro Plus software package.

### Protein Phosphatase activity assay

Cellular protein phosphatase 4 (PP4) activity was assayed using an immunoprecipitation phosphatase assay kit (Millipore). Cells were washed in TBS, and then lysed on ice in phosphatase assay buffer (20mM imidazole-HCl, 2mM EDTA, 2mM EGTA, 0.1% NP-40 (v/v), pH 7.0, with freshly added protease inhibitors). The PP4-C or PP4R2 subunit and the Rabbit IgG control was immunoprecipitated from total cell lysates (0.5-1mg) using 3 μg of anti-PP4-C or anti-PP4R2 antibody (Bethyl), PP4-C and PP4R2 antibody (Bethyl) and Protein A agarose overnight at 4°C. PP4 activity was assayed by incubating the immunoprecipitated protein with the synthetic phosphopeptide K-R-pT-I-R-R at 30°C for 10 min prior to detection with malachite green phosphate detection solution, according to the manufacturer’s instructions. The value associated with IgG immunoprecipitation was subtracted as background. Where indicated, phosphatase activity was normalized to the relative amount of immunoprecipitated C subunit quantified using Image J software (NIH).

### Immunoprecipitation

One 15-cm plate containing cells grown to 80% confluence was used for each treatment condition and divided into aliquots for different immunoprecipitation conditions or antibodies. To harvest, cells were washed twice with PBS on the plate, then lysed on ice using a cell scraper with lysis buffer (150 mM KCl, 0.2% (v/v) NP-40, 10% (v/v) glycerol (v/v), 20 mM Tris pH 7.5, 0.5 mM DTT) containing freshly added protease inhibitor complex (Roche). Following 15 min incubation on ice, lysates were cleared at high speed centrifugation at 14,000 rpm for 10 min before protein quantitation using the BCA assay (Pierce). Equal amounts of lysate (500 μg– 1 mg) were immunoprecipitated while rotating at 4°C with 3 μg of one of the following antibodies: Anti-Flag M2 affinity Gel (Sigma), PP4-C and PP4R2 (Bethyl) or IgG controls: IgG-Mouse or IgG-Rabbit (Sigma). Protein G agarose beads (30 μl) were added to each immunoprecipitate and rotated overnight. Beads were washed four times with lysis buffer, and then resuspended in SDS loading dye and boiled prior to immunoblotting.

### siRNA or shRNA knockdown

On-Target Plus human TIPRL siRNA were purchased from GE Health Care-Dharmacon. HeLa cells were plated to 50–60% confluence and transfected with 20nM siRNA using the RNAi Lipofectamine reagent as recommended (Invitrogen). Knockdown was evaluated 48 hours after transfection. Treatment with CPT in immunoprecipitation reactions were carried out 48hr post transfection. The mouse SM2-shTIPRL retroviral construct was purchased from OpenBiosystems. TIPRL was subcloned into the multiple cloning site of the LMP retroviral vector using the XhoI and EcoRI restriction sites. Stable knockdown cell lines were generated by transfection of 293T cells with retroviral LMP mouse shTIPRL construct using lipofectamine 2000 per recommended conditions. Viral supernatant was collected from transfected cells and the supernatant was cleared by centrifugation at 13,000 rpm. 3T3 MEFs were plated and underwent retroviral transduction by addition of viral supernatant and 10μg/ml polybrene for 4 hours. Cells were replenished with fresh virus and polybrene for an additional 4 hours. 48 hours post transduction, cells were selected with puromycin. The cell population was cloned by limiting dilution and clones were screened for TIPRL knockdown by immunoblotting. Clones with optimal knockdown were used for immunoprecipitation, viability and signaling experiments.

### LPC TIPRL stable cell line generation

TIPRL was PCR amplified from mouse cDNA with the following primers: TIP-F TGC GAA TTC GCC ACC ATG ATG ATC CAC GGC TTT CAG AGC, TIP-R TGC CTC GAG TTA TTC TGA GGG CGT ACT TTG. The TIPRL PCR amplification product was cloned into the multiple cloning site of the LPC FLAG vector using EcoRI and XhoI. 293T cells were transfected with LPC FLAG-TIPRL and viral supernatant was collected and cleared by centrifugation. 3T3 MEF’s were incubated at 37°C for 4 hours with cleared virus containing polybrene (10μg/ml). Virus was replaced after 4 hours, followed by replacement with fresh media. The transduced cells were subjected to puromycin selection. The 3T3 MEFs expressing LPC FLAG TIPRL were cloned by limiting dilution and clones were screened for overexpression. High expressing clones were used in viability, signaling and immunoprecipitation.

## Results

### TIPRL interacts with the PP4 phosphatase and inhibits its activity

**Fig 1:**
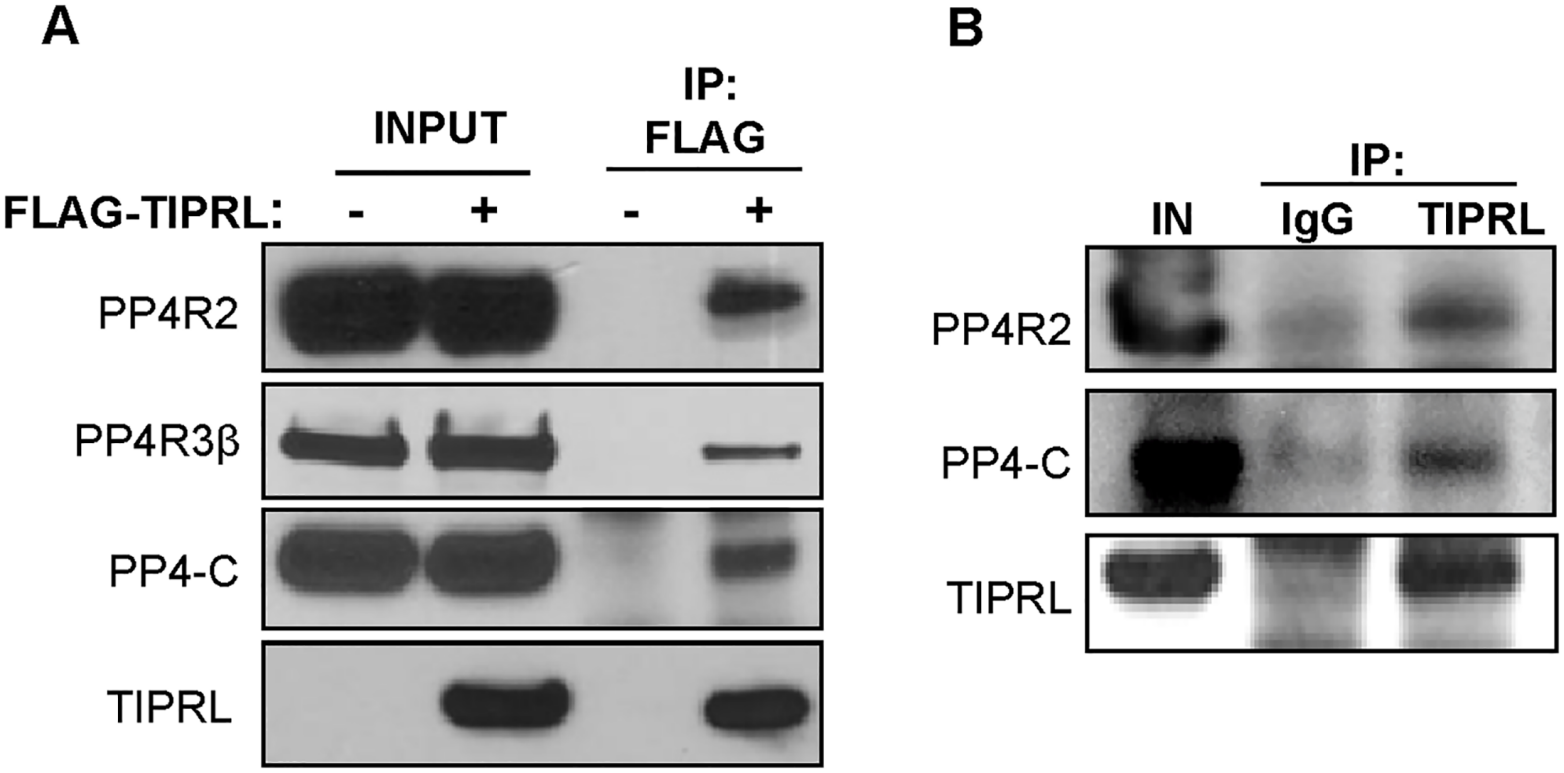
TIPRL interacts with the protein phosphatase 4 complex. (A) 293T cells transfected with LPC FLAG vector (FLAG-TIPRL (-)) or LPC FLAG-TIPRL (FLAG-TIPRL (+)) were immunoprecipitated with FLAG conjugated beads followed by immunoblotting with the indicated antibodies. (B) TIPRL was immunoprecipitated from HeLa cells followed by immunoblotting with the indicated antibodies to assess endogenous interaction with TIPRL.

TIPRL is a highly conserved and ubiquitously expressed protein that interacts with multiple PP2A-family phosphatase catalytic subunits, including PP2A-C, PP4-C and PP6-C. Here, to further investigate if TIPRL also binds with PP4R2, a PP4-C interacting protein that has been found in active PP4 phosphatase complexes, we transiently overexpressed FLAG tagged vector or TIPRL and performed immunoprecipitation followed by immunoblotting. PP4-C and PP4R2 were found to interact with TIPRL (Fig 1A). The endogenous binding of TIPRL with PP4R2 and PP4-C was validated by co-IP experiments (Fig 1B).

**Fig 2:**
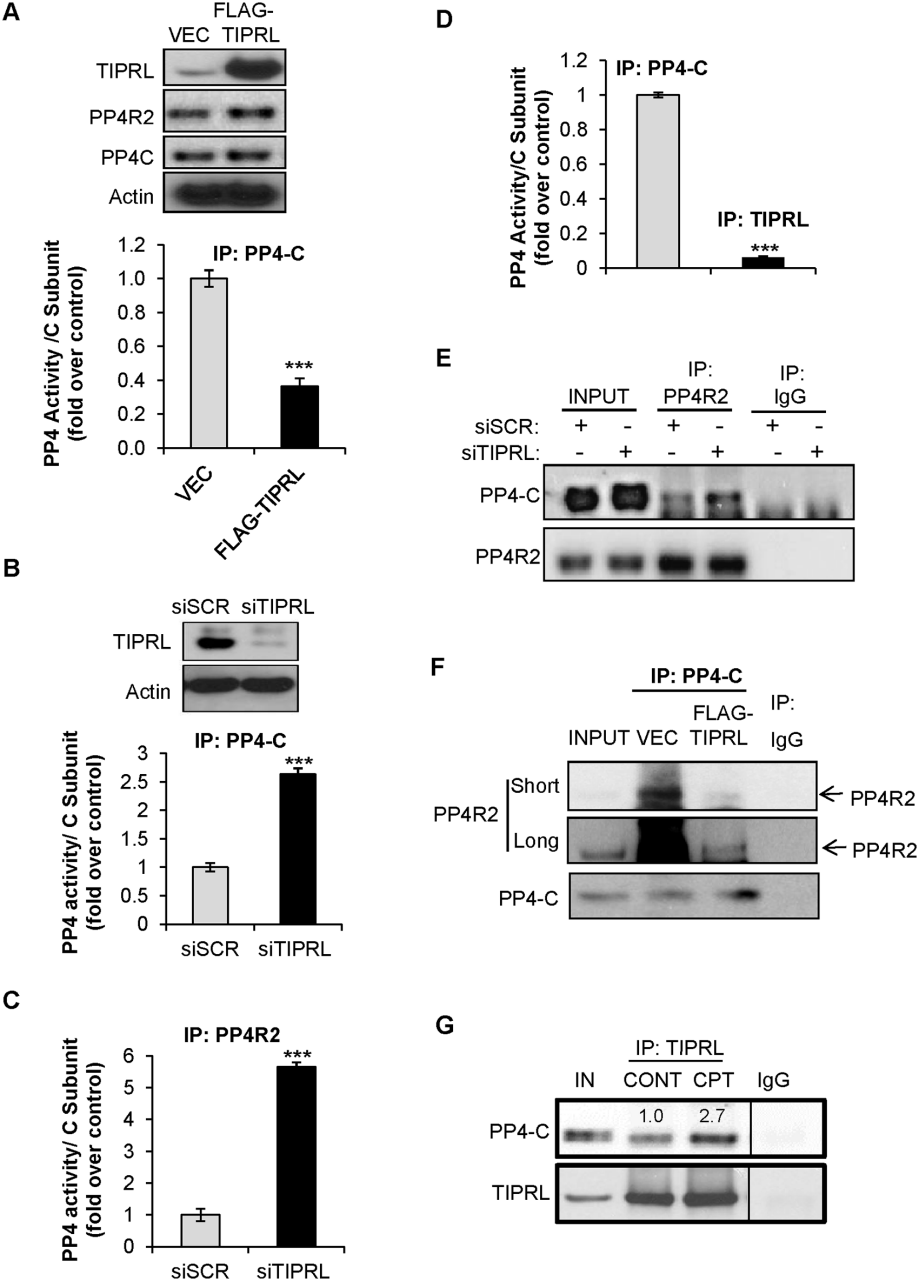
TIPRL inhibits PP4 phosphatase activity and complex assembly. (A) HeLa cells were transiently transfected with LPC FLAG (VEC) or LPC FLAG-TIPRL (FLAG-TIPRL). 24hrs after transfection, cells were lysed and immunoprecipitated with PP4-C antibody, upon which phosphatase activity was measured. Data represent ± standard deviation of the mean of three independent experiments. (B&C) HeLa cells were transfected with a scramble (siSCR) or TIPRL siRNA (siTIPRL). 48hrs after transfection, cell lysates were immunoprecipitated with PP4-C (B) or PP4R2 (C) antibody and phosphatase activity was measured. Data represent ± standard deviation of the mean of three independent experiments. (D) HeLa cells transfected with LPC FLAG-TIPRL (FLAG-TIPRL) were immunoprecipitated with the indicated antibodies and phosphatase activity was measured. (E) HeLa cells were transfected with a scramble (siSCR) or TIPRL siRNA (siTIPRL) and immunoprecipitated with PP4R2 antibody followed by immunoblotting with the indicated antibodies. (F) HeLa cells were transiently transfected with LPC FLAG (VEC) or LPC FLAG-TIPRL (FLAG-TIPRL) and immunoprecipitated with PP4-C antibody followed by immunoblotting with the indicated antibodies. Long and short refer to the film exposure time. (G) 3T3 cells were treated with DMSO (CONT) or 5μM CPT for 1hr. Cells were lysed and immunoprecipitated with TIPRL followed by immunoblotting with the indicated antibodies. Band intensity was quantified using Image ***p<0.001, Student’s t test.

To assess TIPRL’s ability to regulate PP4 protein phosphatase activity, TIPRL was transiently overexpressed in HeLa cells (Fig 2A). Vector and TIPRL overexpressing cell lysates were immunoprecipitated followed by phosphatase activity assays to evaluate the role of TIPRL in regulating phosphatase activity. Immunoprecipitation of the PP4 catalytic subunit in TIPRL overexpressing cells showed a dramatic decrease in phosphatase activity compared to the vector control; however, the total levels of PP4 proteins PP4-C and PP4R2 are unchanged (Fig 2A). To further confirm TIPRL’s role in regulating phosphatase activity, we transiently knocked down TIPRL (Fig 2B) and measured protein phosphatase activity. Immunoprecipitation of the PP4 catalytic subunit displayed a 2.5 fold increase in phosphatase activity when TIPRL was diminished compared to the scrambled control (Fig 2B). As PP4-C resides in both active (i.e. PP4R2/PP4-C complex) and inactive complexes (i.e. α4/PP4-C complex), we next asked if TIPRL preferably inhibits the active PP4 complexes. We immunoprecipitated PP4R2, a PP4-C interacting protein present in active complexes, and subjected the immunoprecipitates to a phosphatase assay and found that knockdown of TIPRL resulted in a 5 fold increase in PP4R2-associated phosphatase activity when PP4R2 was immunoprecipitated (Fig 2C). Compared to the 2.5 fold decrease in total PP4 activity (Fig 2A), this data indicates that TIPRL may target protein phosphatase active complexes. Because TIPRL inhibits phosphatase activity, we next asked whether TIPRL-associated PP4-C was inactive. To test this, we immunoprecipitated PP4-C or TIPRL followed by phosphatase assay. The phosphatase activity was further normalized to the PP4-C subunit detected by western blot in the PP4-C or TIPRL immunoprecipitates. Indeed, compared to total PP4-C activity, TIPRL bound PP4-C was inactive, suggesting TIPRL inhibits PP4-C activity due to direct binding (Fig 2D). Next, we wanted to determine if TIPRL inhibited phosphatase activity by promoting disassembly of the PP4 active complex. The PP4R2/PP4-C complex was evaluated by immunoprecipitating PP4R2 followed by a western blot using anti-PP4-C antibody. Cells expressing a siRNA to TIPRL displayed an increase in PP4-C bound to PP4R2 (Fig 2E), consistent with more phosphatase activity (Fig 2B & 2C). On the other hand, when TIPRL was overexpressed, the amount of PP4-C/PP4R2 complex dramatically decreased compared to vector control (Fig 2F), consistent with the decrease in phosphatase activity under the same conditions (Fig 2A). Furthermore, TIPRL association with PP4-C was increased upon treatment with DNA-damaging agent CPT, suggesting PP4-C is inhibited upon this stress condition via association with TIRPL (Fig 2G). Taken together, these results suggest that TIPRL may inhibit phosphatase activity through promoting disassembly of the active PP4 complex.

### TIPRL promotes γ-H2AX phosphorylation in response to DNA damage

**Fig 3:**
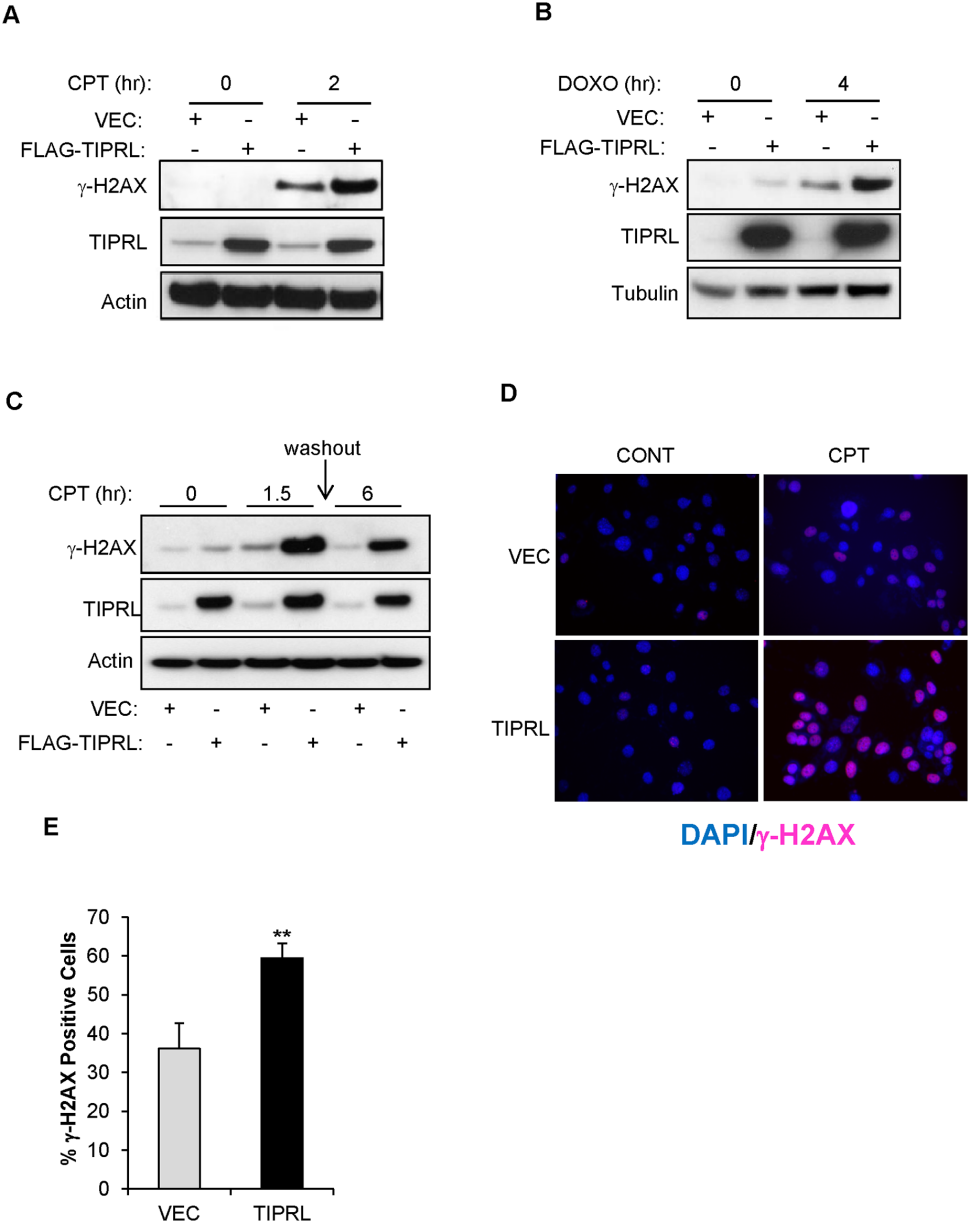
TIPRL promotes γ-H2AX phosphorylation in response to DNA damage. (A) HeLa cells were transiently transfected with vector control (VEC) or FLAG-TIPRL. 24hrs after transfection, cells were treated with CPT (2.5μM) or (B) doxorubicin (DOXO, 2μg/ml) for the indicated time points. Cells were lysed and immunoblots were probed with the indicated antibodies. (C) HeLa cells were transiently transfected with vector control (VEC) or FLAG-TIPRL. 24hrs after transfection, cells were treated with CPT (2.5μM) for 1.5h followed by washout for 6h. Cells were lysed and immunoblots were probed with the indicated antibodies. (D) 3T3 cells stably expressing LPC FLAG vector control (VEC) or LPC FLAG- TIPRL (TIPRL) were treated with 5μM CPT for 1.5hrs. Cells were fixed and stained for both DAPI and γ-H2AX and (E) images were quantified. Data represent ± standard deviation of the mean of three fields**p<0.01, Student’s t test.

The PP4-C/PP4R2/PP4R3β complex has been shown to be a γ-H2AX phosphatase in mammalian cells [3,6] and the *pph3* gene, which encodes the ortholog of PP4 catalytic subunit in budding yeast, serves as the sole γ-H2AX phosphatase in this organism [4,5,7]. We found that TIPRL decreases PP4 activity, therefore we wanted to determine if TIPRL expression also resulted in increased γ-H2AX levels following DNA damage. We transiently overexpressed TIPRL and assessed γ-H2AX levels in response to DNA damage. We found that TIPRL overexpression led to increased γ-H2AX phosphorylation in response to the DNA damaging agents CPT (Fig 3A) and doxorubicin (DOXO) (Fig 3B) compared to the vector control. To determine if TIPRL is involved in regulating γ-H2AX dephosphorylation during recovery from DNA damage, we performed a washout experiment and found, consistent with Fig 3A, TIRPL expression led to increased γ-H2AX compared to vector control cells; however, increased TIPRL levels impaired the ability of cells to reverse DNA damage-induced H2AX phosphorylation following washout of the drug, suggesting a delayed dephosphorylation due to decreased phosphatase acitivity in TIPRL overexpressing cells (Fig 3C). To further investigate the role of TIPRL in promoting γ-H2AX foci in response to DNA damage, TIPRL overexpressing cells were treated with CPT, and γ-H2AX staining was visualized by immunofluorescence microscopy (Fig 3D). TIPRL overexpression resulted in a twenty five percent increase in cells that contained γ-H2AX following CPT treatment (Fig 3E).

**Fig 4:**
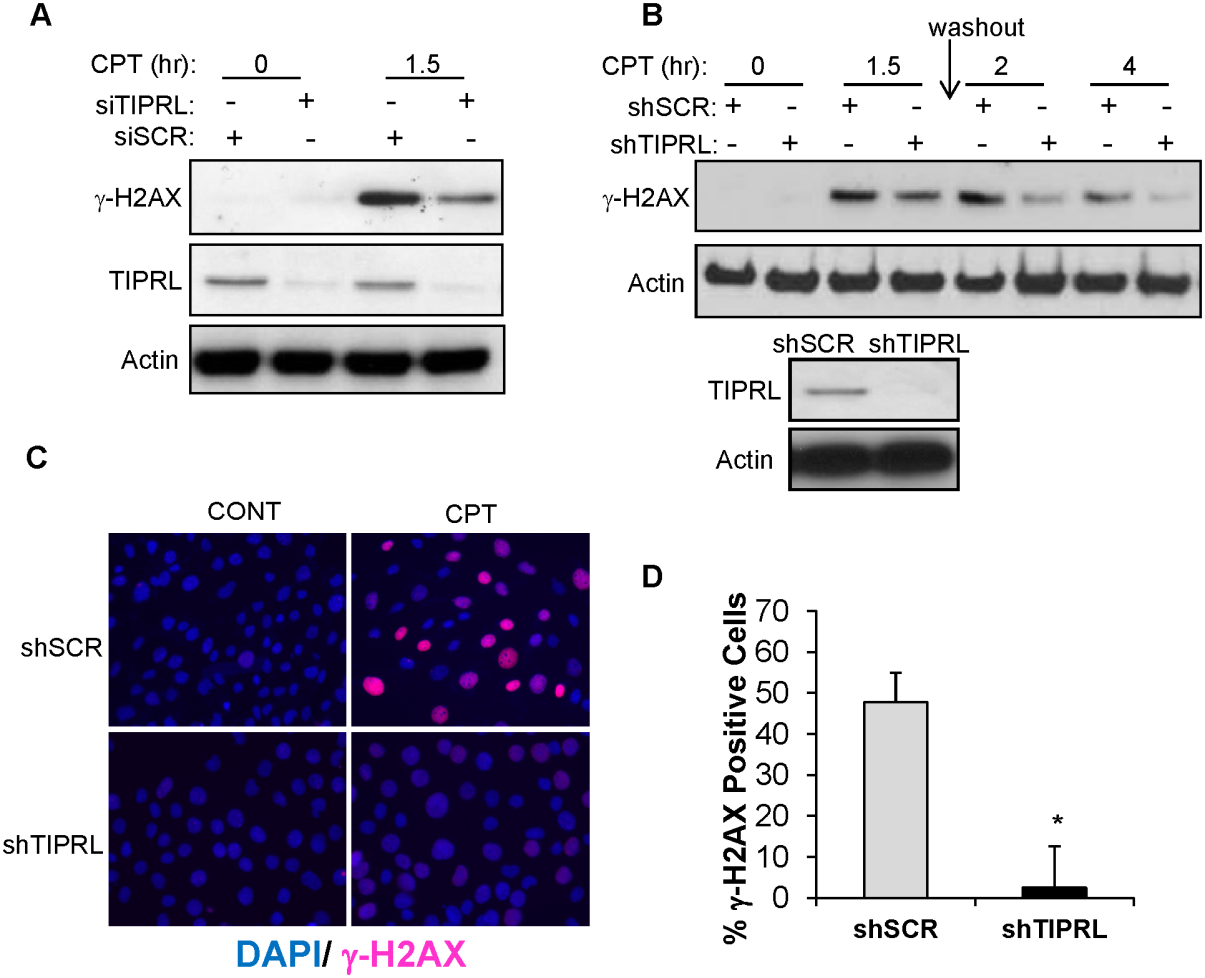
Knockdown of TIPRL inhibits γ-H2AX phosphorylation upon DNA damage. (A) HeLa cells were transfected with a scramble (siSCR) or TIPRL siRNA (siTIPRL). 48hrs after transfection, cells were treated with 2.5μM CPT for 1.5hrs. Cell lysates were prepared and immunoblotting was performed using the indicated antibodies. (B) 3T3 MEFs infected with retrovirus containing a short hairpin (sh) against TIPRL (shTIPRL) or scrambled shRNA (shSCR) were treated with 2.5μM CPT for 1.5hrs. The drug was washed out of the cells and fresh media was added back for the indicated amount of time. Cell lysates were prepared and immunoblots were probed with the indicated antibodies. (C) 3T3 MEFs expressing a short hairpin (sh) against TIPRL (shTIPRL) or scrambled shRNA (shSCR) were treated with 5μM CPT for 1hr and stained with both DAPI and anti-γ-H2AX (D) followed by quantification of the images. Data represent ± standard deviation of the mean of three fields. *p<0.01, Student’s t test.

To further investigate the role of TIPRL in mediating γ-H2AX levels in response to DNA damage, TIPRL was transiently knocked down followed by treatment with CPT. Decreased TIPRL levels resulted in a decrease in γ-H2AX phosphorylation compared to scrambled control in response to DNA damage (Fig 4A). To determine if TIPRL is involved in regulating γ-H2AX de-phosphorylation during recovery from DNA damage, cells expressing stable knockdown of TIPRL were generated (Fig 4B). Cells expressing shTIPRL were treated with the DNA damaging agent CPT for 1.5 hours and the drug was washed out allowing cells to recover for the indicated time. Decreased TIPRL levels enhanced the ability of cells to reverse DNA damage-induced H2AX phosphorylation following washout of the drug (Fig 4B). In addition, knockdown of TIPRL resulted in a forty percent decrease in γ-H2AX positive foci in response to DNA damage (Fig 4C & 4D). From these results we conclude that TIPRL promotes γ-H2AX phosphorylation in response to DNA damage.

### TIPRL promotes cell death in response to genotoxic stress

**Fig 5:**
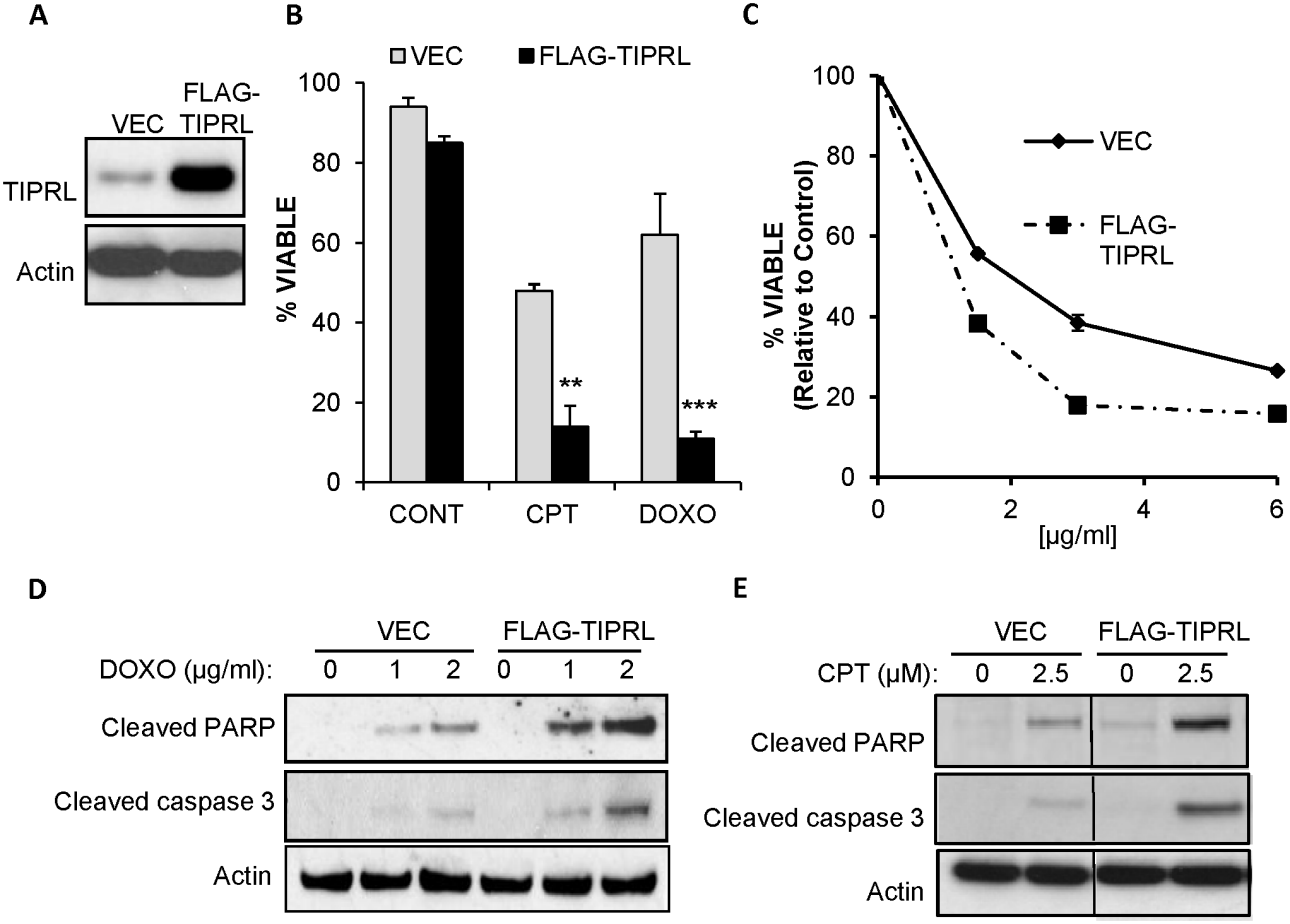
Overexpression of TIPRL promotes cell death in response to genotoxic stress. (A) 3T3 MEFs stably expressing LPC FLAG vector control (VEC) or LPC FLAG-TIPRL (FLAG-TIPRL) were lysed and immunoblotted with the indicated antibodies. (B) Cells were treated with 10μM CPT or 2μg/ml doxorubicin for 24hrs. Viability was measured by propidium iodide exclusion. Data represent ± standard deviation of the mean of three independent experiments. (C) 3T3 MEFs stably expressing LPC FLAG vector control (VEC) or LPC FLAG-TIPRL (FLAG-TIPRL) were treated with the indicated concentration of doxorubicin (DOXO) for 24hrs. Cell viability was measured by MTS assay. (D) 3T3 cells stably expressing LPC FLAG vector control (VEC) or LPC FLAG-TIPRL (FLAG-TIPRL) were treated with the indicated concentration of doxorubicin (DOXO) for 24hrs. Cells were lysed and immunoblotted with the indicated antibodies. (E) 3T3 MEFs stably expressing LPC FLAG vector control (VEC) or LPC FLAG-TIPRL (FLAG-TIPRL) were treated with the indicated concentration of CPT for 24hrs. Cells were lysed and immunoblotted with the indicated antibodies. **p<0.005, ***p<0.001, Student’s t test.

Persistent stress signaling is linked to the initiation of apoptosis in mammals. We found that TIPRL promotes hyperphosphorylation of γ-H2AX in response to DNA damage (Fig 3). Next, to further determine if TIPRL expression sensitizes cells to genotoxic stress, cells stably overexpressing TIPRL (Fig 5A) were treated with genotoxic agents. We found that elevated TIPRL protein levels resulted in increased sensitivity to both DNA damaging agents CPT and doxorubicin (DOXO) (Fig 5B). In addition, a dose response experiment further validated the effect of TIPRL in promoting cell death upon drug treatments (Fig 5C). To determine if the cells were dying through apoptosis, PARP and caspase 3 cleavages were evaluated. Consistent with the increase in cell death, overexpression of TIPRL resulted in increased cleaved PARP and caspase 3 in response to both DOXO (Fig 5D) and CPT (Fig 5E) treatments, suggesting that TIPRL plays an important role in mediating apoptotic cell death in response to genotoxic stress.

**Fig 6:**
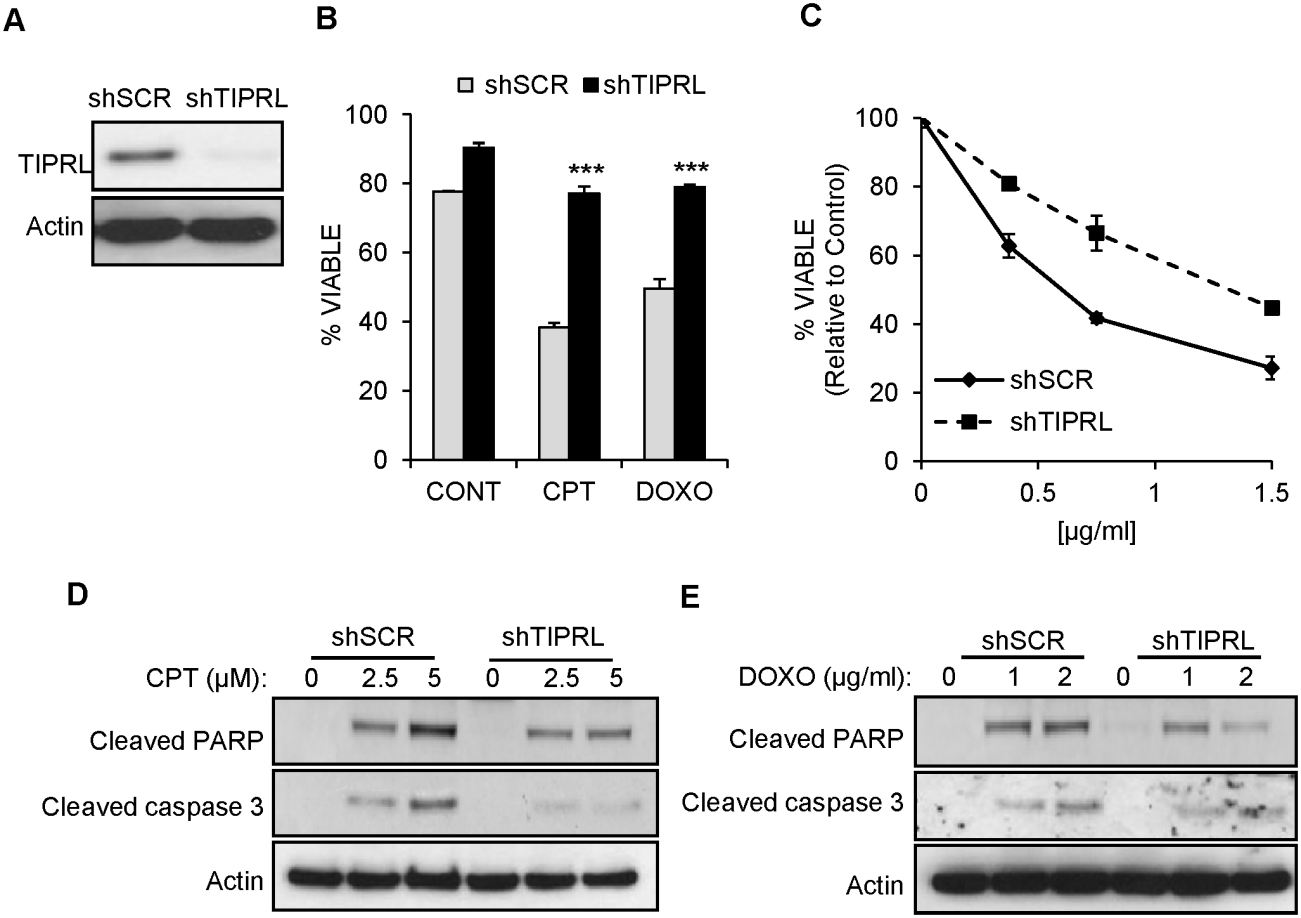
Knockdown of TIPRL protects from cell death in response to genotoxic stress. (A) 3T3 MEFs expressing a short hairpin against scramble (shSCR) or TIPRL (shTIPRL) were immunoblotted with the indicated antibodies. (B) 3T3 MEFs expressing a short hairpin against scramble (shSCR) or TIPRL (shTIPRL) were treated with 10μM CPT or (2μg/ml) doxorubicin (DOXO) for 24hrs. Cell viability was measured using propidium iodide exclusion. Data represent ± standard deviation of the mean of three independent experiments. (C) 3T3 MEFs expressing a short hairpin against scramble (shSCR) or TIPRL (shTIPRL) were treated with the indicated amounts of doxorubicin (DOXO) for 24hrs. Viability was determined by MTS assay. (D) 3T3 MEFs expressing a short hairpin against scramble (shSCR) or TIPRL (shTIPRL) were treated with the indicated amounts of CPT or (E) doxorubicin (DOXO) for 24hrs and PARP and caspase 3 cleavages were evaluated by immunoblotting. ***p<0.001, Student’s t test.

To further determine if knockdown of TIPRL protects from cell death in response to genotoxic stress, TIPRL knockdown cells (Fig 6A) were evaluated for their sensitivity to the DNA damaging agents CPT and DOXO. When TIPRL levels were decreased, this resulted in dramatic protection from cell death in response to genotoxic stress (Fig 6B). In addition, TIPRL knockdown cells are more resistant to the DNA damaging agents, DOXO, in a dose dependent manner (Fig 6C). Consistent with the decrease in cell death (Fig 6B), TIPRL knockdown cells displayed less cleaved PARP and cleaved caspase 3 in response to both CPT (Fig 6D) and DOXO (Fig 6E) treatment. These results indicate that TIPRL plays an important role in cell death in response to DNA damage.

## Discussion

Aberrant protein phosphorylation has been linked to many diseases, including cancer. Unlike kinases, the role and regulation of protein phosphatases in disease and therapeutic response has not been well established. Here we show that TIPRL, an evolutionarily conserved protein, plays a critical role in mediating γ-H2AX signal transduction upon DNA damage. We found that TIPRL’s function in stress responses is conserved. In yeast, overexpression of TIP41, TIPRL ortholog, caused a severe growth defect, and deletion of TIP41 conferred partial resistance to rapamycin [9]. Our studies demonstrated that, in the mammalian system, TIPRL displays a conserved phenotype in stress responses, as overexpression of TIPRL sensitized cells to DNA damage (Fig 5), while knockdown of TIPRL protected cells from this stress (Fig 6).

How is TIPRL mediating H2AX phosphorylation and cell survival in response to DNA damage? Previous studies have elucidated the role for PP4 in acting as the phosphatase for γ-H2AX and loss of PP4-C resulted in sensitization to DNA damaging agents CPT and hydroxyurea [3,6]. Consistently, we found that TIPRL acts as an inhibitor of PP4 phosphatase, which leads to enhanced H2AX phosphorylation and cell sensitivity to DNA damaging agents. Knockdown of TIPRL increased the amount of PP4-C/PP4R2 complex available indicating that, as TIPRL levels decrease, PP4-C and PP4R2 are bound less to TIPRL and more available to form into the active γ-H2AX phosphatase complex [6]. We didn’t find a significant increase in TIPRL protein level upon CPT treatment (Figs 3A, 3C & 4A). Instead, we found an increase in TIRPL and PP4-C complex upon CPT treatment (Fig 2G). These data suggest that TIPRL achieves inhibition of the γ-H2AX phosphatase complex in response to DNA damage by binding to PP4R2 and PP4-C, holding them in an inactive state. Interestingly, TIPRL levels have been shown to influence the phosphorylation state of a protein substrate of ataxia-telangiectasia mutated (ATM) and Rad3 related (ATR) kinases using ATM/ATR specific substrate antibodies [12]. In this paper, elevated levels of TIPRL resulted in an increase in the phosphorylation state of this protein, while TIPRL depletion decreased the phosphorylation state of this protein, indicating that TIPRL may act as an inhibitory regulator of phosphatase complexes that mediate de-phosphorylation of signaling proteins within the ATM/ATR pathway that control DNA replication and repair [12]. This study correlates with our finding that TIPRL promotes phosphorylation of H2AX via inhibiting phosphatase activity. Because PP6 and WIP1 reportedly dephosphorylate γ-H2AX in certain contexts, we are unable to exclude that TIPRL may also affect γ-H2AX through mechanisms independent of PP4 [14,15].

Our data provides insight into the mechanisms that regulate protein phosphatase activity in response to DNA damage. Currently, chemotherapeutic options that utilize DNA damaging agents are still widely used; however, resistance to these commonly used agents is becoming a substantial hurdle in effectively treating patients. Interestingly, PP4 has been shown to be overexpressed in human breast and lung tumors [16]. It will be interesting to further determine the TIPRL status of these human tumors and whether loss of TIPRL contributes to PP4-C overexpression and the clinical implication this has on chemoresistance. Clearly, understanding the mechanisms that regulate protein phosphatase activity to maintain cellular homeostasis may lead to novel drug targets able to combat aggressive chemoresistant tumors.

TIPRL is a conserved binding protein to all of the PP2A-family phosphatases, including PP2A, PP4 and PP6 [10,11], which is similar to α4, another global regulator of PP2A-family phosphatases. Thus, in the future, it will be interesting to further determine if TIPRL acts as a global inhibitor for all of the phosphatases it binds to, and whether it regulates multiple signaling pathways, which will help to elucidate whether TIPRL regulation is specific to the DNA damage response.
